# No Genetic Influence for Childhood Behavior Problems From DNA Analysis

**DOI:** 10.1016/j.jaac.2013.07.016

**Published:** 2013-10

**Authors:** Maciej Trzaskowski, Philip S. Dale, Robert Plomin

**Affiliations:** a Social, Genetic, and Developmental Psychiatry Centre, Institute of Psychiatry, King's College London; b University of New Mexico

**Keywords:** behavior problems, cognitive abilities, genome-wide complex trait analysis (GCTA), heritability, twin study

## Abstract

**Objective:**

Twin studies of behavior problems in childhood point to substantial genetic influence. It is now possible to estimate genetic influence using DNA alone in samples of unrelated individuals, not relying on family-based designs such as twins. A linear mixed model, which incorporates DNA microarray data, has confirmed twin results by showing substantial genetic influence for diverse traits in adults. Here we present direct comparisons between twin and DNA heritability estimates for childhood behavior problems as rated by parents, teachers, and children themselves.

**Method:**

Behavior problem data from 2,500 UK-representative 12-year-old twin pairs were used in twin analyses; DNA analyses were based on 1 member of the twin pair with genotype data for 1.7 million DNA markers. Diverse behavior problems were assessed, including autistic, depressive, and hyperactive symptoms. Genetic influence from DNA was estimated using genome-wide complex trait analysis (GCTA), and the twin estimates of heritability were based on standard twin model fitting.

**Results:**

Behavior problems in childhood—whether rated by parents, teachers, or children themselves—show no significant genetic influence using GCTA, even though twin study estimates of heritability are substantial in the same sample, and even though both GCTA and twin study estimates of genetic influence are substantial for cognitive and anthropometric traits.

**Conclusions:**

We suggest that this new type of “missing heritability,” that is, the gap between GCTA and twin study estimates for behavior problems in childhood, is due to nonadditive genetic influence, which will make it more difficult to identify genes responsible for heritability.

Behavior problems in childhood, such as anxiety, depression, autistic symptoms, hyperactivity, and conduct problems, are common, with a cumulative incidence during childhood of 12% for 1 or more disorders.^1^ They are not always transient problems that disappear as children develop: one-half of all lifetime cases of diagnosed psychopathology begin in childhood.^2^

Furthermore, their heritability is surprisingly high: for example, twin studies using parent ratings typically report heritabilities in the range of 40% for anxiety and depression to 60% for autistic symptoms and hyperactivity.^3^ Consequently, childhood behavior problems have become the target of genome-wide association (GWA) studies that attempt to identify the genes responsible for their heritability. As in other life sciences,^4^ these GWA expeditions have come up largely empty-handed.^5^ This ‘missing heritability’ is the key puzzle in DNA research on complex traits and common disorders—only a small portion of genes responsible for their heritability has been identified.^5^, ^6^ Although attention has focused on the difficulties in identifying the many genes of small effect responsible for heritability, the other side of the missing heritability gap is the heritability estimate itself, which has relied on family-based studies of twins and adoptees.^3^

It is now possible to test the validity of these heritability estimates using DNA from unrelated individuals, a method called genome-wide complex trait analysis (GCTA).^7^, ^8^ GCTA research has shown that the common DNA variants (single-nucleotide polymorphisms [SNPs]) genotyped on DNA arrays used in GWA studies yield substantial estimates of genetic influence for height^7^ and weight,^9^ psychiatric and medical disorders,^10^, ^11^, ^12^ personality,^13^ and cognitive traits.^14^, ^15^, ^16^ However, these GCTA estimates of genetic influence cannot completely close the heritability gap, in part because GCTA is limited to additive effects of causal variants tagged by the common SNPs on current DNA arrays used in GWA research.^8^

For the first time, we report GCTA estimates for childhood behavior problems as rated by parents, teachers, and the children themselves. To gauge the true breadth of the missing heritability gap, we compared GCTA estimates to twin heritability estimates for the same measures in the same sample. We also compared these results for behavior problems to results for height and weight, and cognitive traits in the same sample.

## Method

### Sample and Genotyping

The sample was drawn from the Twins Early Development Study (TEDS), which is a multivariate longitudinal study that recruited more than 11,000 twin pairs born in England and Wales in 1994, 1995, and 1996.^17^ TEDS has been shown to be representative of the UK population.^18^ The project received approval from the Institute of Psychiatry ethics committee (05/Q0706/228), and parental consent was obtained before data collection.

The present analyses were limited to children for whom DNA, genome-wide genotyping, and behavior problem and cognitive data were available. Moreover, the twin analyses were based only on twins included in the GCTA analyses, to provide a more precise comparison between GCTA and twin study results.

DNA was available for 3,747 children 11 and 12 years of age (mean age, 11.5 years) whose first language was English and who had no major medical or psychiatric problems. From that sample, DNA samples of 3,665 individuals (only 1 member of a twin pair) were successfully hybridized to Affymetrix GeneChip 6.0 SNP genotyping arrays using standard experimental protocols as part of the WTCCC2 project. In addition to nearly 700,000 genotyped SNPs, more than 1 million other SNPs were imputed from HapMap 2, HapMap 3, and WTCCC controls, using IMPUTE v.2 software.^19^ A total of 3,152 DNA samples (from 1,446 males and 1,706 females) survived quality control criteria for ancestry, heterozygosity, relatedness, and hybridization intensity outliers. To control for ancestral stratification, we performed principal component analyses on a subset of 100,000 quality-controlled SNPs after removing SNPs in linkage disequilibrium (r^2^ > 0.2).^20^ Using the Tracy-Widom test,^21^ we identified 8 axes with *p* < .05, which were used as covariates in GCTA analyses.

Of these 3,152 children, the present analyses were limited to those for whom behavior problem and cognitive data were available. Twin zygosity was diagnosed on the basis of physical similarity, and questionable cases were verified with analysis of DNA markers.^18^ As expected, approximately equal numbers of monozygotic (MZ), dizygotic (DZ) same-sex, and DZ opposite-sex twins were included; DZ same-sex and opposite-sex pairs were combined to increase power and because previous twin analyses of these data show no evidence of qualitative or quantitative sex differences in sex-limitation models.^22^ For the measures of behavior problems, the numbers of individuals for GCTA analyses range from 2,687 to 2,698 for self-report, 2,687 to 2,700 for parent ratings, and 2,034 to 2,139 for teacher ratings. The numbers of pairs of twins range from 2,668 to 2,683 for self-report, 2,680 to 2,695 for parent ratings, and 1,783 to 1,925 for teacher ratings. The sample sizes for the GCTA results shown are 2,325 for “g” and language, 2,238 for “g” and mathematics, 2,250 for “g” and reading, and 2,296 for height and weight.

### Measures

All of the measures have been reported in previous TEDS publications, which can be consulted for greater detail.^23^

#### Behavior Problems

The behavior problem measures described in this section have been widely used in the literature. As is the case in the literature, these measures are modestly correlated, 0.33 on average for the scores described below. Given the modest correlation among the measures and the focus of the present paper on comparisons between GCTA and twin estimates for diverse behavior problems, we present results separately for the behavior problem scales rather than conducting multivariate analyses.

#### Conners (Attention-Deficit/Hyperactivity [ADHD])

ADHD symptoms were assessed via parent-rated questionnaire, which was the *DSM-IV*–based ADHD scale from the Conners' Parent Rating Scale–Revised (CPRS-R). The questionnaire consisted of 2 scales: inattentiveness and hyperactivity-impulsivity.

#### Antisocial Process Screening Devise (APSD; Psychopathic Symptoms)

Antisocial behavior was assessed using parent and teacher ratings on the Antisocial Process Screening Device. The questionnaire included 3 scales: callous-unemotional, impulsivity, and narcissism.

#### Childhood Asperger Syndrome Test (CAST; Autistic-Like Symptoms)

The Childhood Asperger Syndrome Test questionnaire was rated by parents and teachers and includes 3 scales: communication, nonsocial, and social, from which a composite was also formed.

#### Moods and Feelings Questionnaire (MFQ; Depressive Symptoms)

The Moods and Feelings Questionnaire was rated by the children and their parents.

#### Strengths and Difficulties Questionnaire (SDQ; Behavior Problems)

The Strengths and Difficulties Questionnaire was assessed by the children and their parents. The questionnaire includes 4 behavior problem scales (anxiety, conduct, hyperactivity, peer problems) from which a composite was created. The SDQ also includes a positive prosocial scale that was not included in these analyses of behavior problems.

#### Cognitive Tests

Cognitive data were collected online via the Internet using adaptive branching, which enabled measurement of the full range of ability using a relatively small number of items.

#### Reading

Four measures of reading were used. Two measures assessed reading comprehension: the reading comprehension subtest of the Peabody Individual Achievement Test (PIAT) and the Global Online Assessment for Learning (GOAL) Formative Assessment in Literacy for Key Stage 3. Reading fluency was assessed by an adaptation of the Woodcock-Johnson III Reading Fluency Test (WJRF) and by the Test of Word Reading Efficiency (TOWRE), which was administered by telephone.

#### Mathematics

Assessment of mathematics targeted 3 components of mathematics: understanding numbers, non-numerical processes, and computation and knowledge. The items for these 3 scales were based on the National Foundation of Educational Research 5–14 Mathematics Series.

#### Language

Three components of language were assessed: syntax, semantics, and pragmatics. Syntax was measured using the Listening Grammar subtest of the Test of Adolescent and Adult Language. Semantics was assessed using Level 2 of the Figurative Language subtest of the Test of Language Competence. Pragmatics was assessed using Level 2 of the Making Inferences subtest of the Test of Language Competence.

#### Verbal, Nonverbal, and General Cognitive Abilities

The verbal tests were Wechsler Intelligence Scale for Children as a Processing Instrument (WISC-III-PI) Multiple Choice Information (General Knowledge) and Vocabulary Multiple Choice subtest. The 2 nonverbal reasoning tests were Wechsler Intelligence Scale for Children–III (WISC-III-UK) Picture Completion and Raven's Standard and Advanced Progressive Matrices. General cognitive ability was indexed as a composite of the 4 verbal and nonverbal tests.

#### Height and Weight

Height and weight were assessed at age 12 years via self-report.

#### Composite Measures

To create composite scores for the measures, standardized residuals were derived for each scale regressed on sex and age. Outliers above or below 3 SD from the mean were excluded and the scale was quantile normalized.^24^, ^25^ The composites were created as unit-weighted means requiring complete data for more than half of the measure's scales (i.e., 3 of 4 or 2 of 3 scales). All procedures were executed using R (www.r-project.org^26^).

### Statistical Analyses

#### Genome-Wide Complex Trait Analysis (GCTA)

GCTA software was used to conduct these analyses.^8^ Of note, although GCTA is a software package, for simplicity we will refer to the full process of estimating genetic influence from SNP data simply as GCTA. Before the variance of a trait can be decomposed, the first step is to calculate pairwise genomic similarity between all pairs of individuals in the sample using all genetic markers genotyped or imputed from the SNP array. Because GCTA is designed to estimate genetic variance due to close linkage disequilibrium between unknown causal variants and genotyped SNPs from a sample of unrelated individuals in the population, any close genetic relatedness is eliminated; for this reason, any individual whose genetic similarity is equal to or greater than a third or fourth cousin is removed (estimate of pairwise relatedness > 0.025).

Conceptually, when performing GCTA analysis, we compare a matrix of pairwise genomic similarity to a matrix of pairwise phenotypic similarity using a random-effects mixed linear model.^7^ In univariate analysis, the variance of a trait can be partitioned using residual maximum likelihood into genetic and residual components. Therefore the residual component includes any source of variance that is not an additive effect of common SNPs, including nonadditive genetic effects, rare variants, environment, gene–environment interaction, and error. Detailed description of this method can be found in Yang *et al.*^7^, ^8^ The 8 principal components described earlier were used as covariates. As mentioned in the previous section, all phenotypes were age and sex regressed before analysis.

#### Twin Analysis

In contrast to GCTA, the twin method models variance/covariance for pairs of related individuals: monozygotic (MZ) twins who are genetically 100% identical, and dizygotic (DZ) twins who share on average 50% of their segregating alleles. Differences in within-pair correlations for MZ and DZ twins are then used to partition the variance into genetic and environmental effects. Variance is attributed to additive genetic influence to the extent that MZ correlations are higher than those for DZ twins. The twin method, unlike GCTA, partitions environmental influence into 2 components: shared or common (C) environmental influences, which is residual MZ twin resemblance not explained by genetics, and nonshared or unique environmental influences (E), which is the extent to which MZ twins differ and includes error of measurement. Detailed description of this A (additive genetics) C (common environment) and E (unique environment; ACE) model and the discussion of related issues can be found elsewhere.^3^

The twin data were modeled using Cholesky decomposition of the variance within structural equation modeling software OpenMx.^27^ Standard univariate model-fitting procedures were followed, as described in previous TEDS publications^18^; standard errors were derived from 95% CI. In most cases, the influence of shared environment was not significant; however, the full ACE model was used for the comparison with the GCTA results. Although C was not significantly different from zero, dropping it from the model could have inflated A, which would have confounded the comparison between twin and GCTA results. It should be noted that GCTA does not discriminate C and E; both C and E are included in the GCTA estimate of nongenetic residuals. In other words, the “A” and “E” of GCTA and the “A” and “E” of twin ACE model fitting are not the same. For GCTA, “A” denotes additive effects of DNA variants tagged by the common SNPs on our DNA array, and “E” includes all residual variance. In contrast, in twin analysis, “A” includes additive genetic effects of any DNA sequence differences, not just common SNPs; variance not explained by A is partitioned into C and E.

## Results

**Figure 1:**
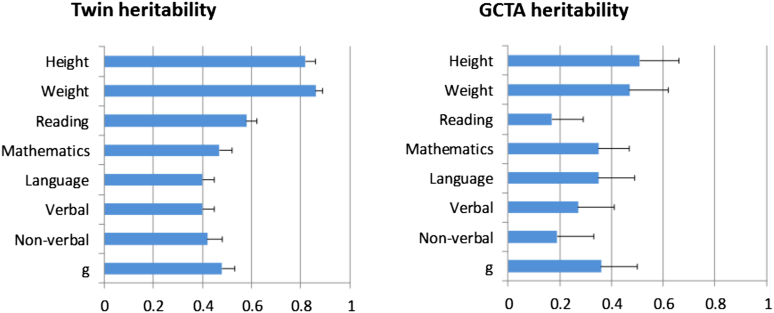
Genetic estimates for height, weight, and cognitive trait composites from twin analyses and from genome-wide complex trait analysis (GCTA). Note: ‘g’ refers to general cognitive ability, which is a composite of verbal and nonverbal ability. N = 2,153 to 2,659 twin pairs for twin analyses, and N = 2,281 to 2,809 unrelated individuals for GCTA. Error bars in the figure indicate standard errors (SE).

Figure 1 compares GCTA and twin study estimates for the anchor variables of height and weight, as well as for cognitive traits. These results are based on the same individuals and twin pairs used in the present analyses of behavior problems, although the results are highly similar to those previously published for the entire TEDS sample.^16^ As expected from the literature, the twin study heritability estimates for height and weight are about 80% and the estimates for the cognitive traits are about 50% (∼40%–60%). The GCTA estimates are about 40% for height and weight and about 25% (∼20%–30%) for the cognitive traits. All of the GCTA estimates are statistically significant, as indicated by the standard errors. These significant and substantial GCTA estimates have 2 important implications. First, they validate the twin method. Second, they imply that sufficiently large GWA studies using current DNA arrays limited to additive effects of common SNPs should be able to account for about 50% of the heritability for height, weight, and cognitive traits. The finding that GCTA estimates are only one-half of the twin heritability estimates is similar to previous reports for these variables and could be due to several factors that either result in GCTA underestimates of twin heritability, such as nonadditive gene–gene interactions, gene–environment interactions, and rare alleles, or to factors that lead to inflation of heritability estimates in twin studies.^5^

**Figure 2:**
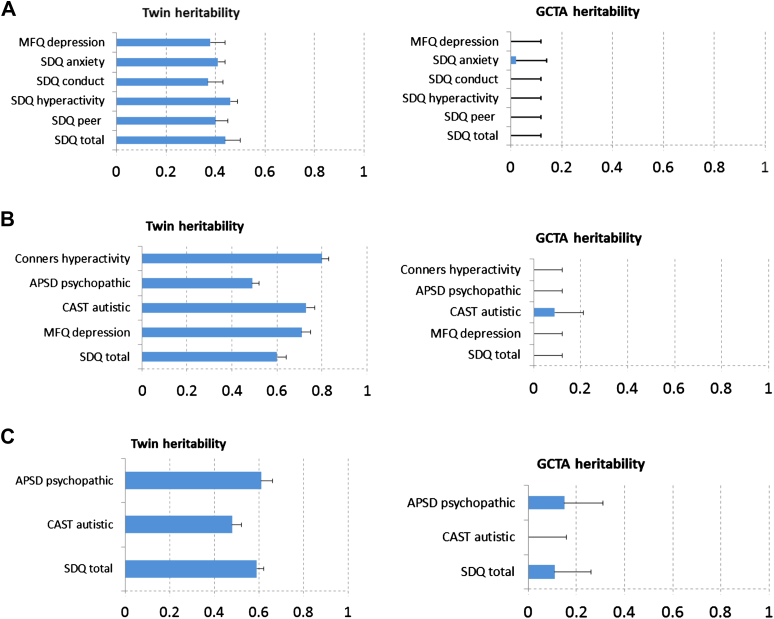
Genetic estimates for composite measures of behavior problems from twin analyses and from genome-wide complex trait analysis (GCTA). Note: (A) Self-report, N = 2,153 to 2,659 twin pairs for twin analyses; N = 2,281 to 2,809 unrelated individuals for GCTA. (B) Parent ratings, N = 2,680 to 2,695 twin pairs for twin estimates; N = 2,687 to 2,700 individuals for GCTA estimates. (C) Teacher ratings, N = 1,783 to 1,925 twin pairs for twin analyses; N = 2,034 to 2,139 individuals for GCTA estimates. Error bars in the figure indicate standard errors (SE). Results for the constituent scales for these composites are presented in Table S1, available online.

Figure 2 compares GCTA and twin study estimates of heritability for composite measures of behavioral problems for self-report, parent ratings, and teacher ratings. Results for the scales that comprise these composites as well as the full variance decomposition are included in Table S1, available online. Twin heritability estimates are similar to those reported in the literature, that is, about 40% heritability for self-report and about 60% heritability for parent and teacher ratings. In contrast, GCTA estimates are nonsignificant and mostly zero for self-report and parent measures of behavior problems. For teacher ratings, a hint of genetic influence emerged, although these GCTA estimates of about 10% are not nearly statistically significant, as indicated by the standard errors. The standard errors are larger for GCTA estimates than for twin estimates because GCTA is based on slight (<2.5%) overall pair-by-pair differences in genetic similarity across the 1.7 million SNPs genotyped from the DNA array, whereas the twin estimate is based on the comparison of 100% genetic similarity for MZ twins and 50% similarity for DZ twins for additive genetic effects. However, if the GCTA estimates for behavior problems were one-half of the twin estimates of heritability, as in the case of height and weight and cognitive traits (Figure 1), the GCTA analysis would have adequate power to detect them, as indicated by the standard errors.

## Discussion

Why do GCTA estimates show no significant genetic influence for diverse childhood behavior problems as rated by parents, teachers, or children themselves, even though twin study estimates of heritability are significant and substantial in the same sample using the same measures, and even though GCTA estimates for cognitive traits are significant and substantial? One broad category of explanations involves mechanisms by which GCTA underestimates twin heritability, more so for behavior problems than for cognitive traits. As mentioned earlier, GCTA underestimates twin heritability because it captures only additive genetic effects tagged by the common SNPs used on GWA arrays. Gene–gene interactions, gene–environment interactions, and rare alleles will widen the gap between GCTA and twin estimates of heritability. However, it is not clear why this gap would be greater for behavior problems than for cognitive traits.

Nonetheless, we can test 1 of these hypotheses in our study, namely, that nonadditive genetic variance is greater for behavior problems than for cognitive traits. Because MZ twins are identical genetically, they are identical even for interactions among many genes (epistasis), whereas such epistatic effects, on average, scarcely contribute to similarity for DZ twins.^3^ Additive genetic effects contribute to MZ twins being twice as similar as DZ twins, whereas the hallmark of nonadditive genetic variance is that MZ twins are more than twice as similar as DZ twins. However, in our study there is no evidence for nonadditive genetic influence either for behavior problems or for cognitive traits: In all cases, MZ correlations are no more than twice as similar as DZ twins. For example, for total behavior problems, MZ and DZ correlations are 0.56 and 0.32, respectively, for self-reports in adolescence; 0.80 and 0.52 for parent ratings; and 0.61 and 0.31 for teacher ratings. Nonetheless, as discussed later, it is possible that nonadditive genetic effects for behavior problems are masked by other factors.

Another, nonmutually exclusive, explanation of why GCTA underestimates twin heritability for behavior problems more than for cognitive abilities is that additive genetic variance might be greater for cognitive traits than for behavior problems. Not previously considered in this context is assortative mating, which increases additive genetic variance for all loci associated with the trait for which spouse correlate. Assortative mating, indexed by the correlation between spouses, is often greater for cognitive traits than for behavior problems: spouse correlations are about 0.40 for cognitive ability.^28^ In contrast, assortative mating was reported to be 0.00 for autistic symptoms^29^ and 0.02 for hyperactivity in 1 study,^29^ and 0.38 and 0.11, respectively, in other studies.^30^, ^31^ Because assortative mating is trait specific and increases additive genetic variance cumulatively over generations, the greater assortative mating for cognitive traits than for behavior problems would increase additive genetic variance for cognitive traits but not for behavior problems. Because GCTA detects only additive genetic variance, assortative mating could thus account for the higher GCTA estimates for cognitive traits than for behavior problems. An obstacle to this hypothesis is that more additive genetic variance for cognitive traits than for behavior problems should lead to higher heritabilities for cognitive traits; however, the results in Figures 1 and 2 show that this is not the case, although this effect could also be masked by countervailing effects, as discussed later.

A second broad category of explanations, and again not mutually exclusive, is that twin studies overestimate heritability for behavior problems more than for cognitive traits. One reason to take this seriously is that twin studies yield higher estimates of heritability than do adoption studies for personality traits, which are related to behavior problems in that personality includes traits such as emotionality, impulsivity, and activity level.^32^ Moreover, the first report of GCTA estimates for personality supported the adoption results with estimates of about 10%, the lowest reported GCTA estimates for any domain of behavior before the present study of behavior problems.^13^ In contrast, heritability estimates are similar for twin and adoption studies of cognitive traits.^5^ It has been suggested that the twin/adoption heritability difference for personality is due to greater nonadditive genetic variance for personality than for cognitive traits, because estimates of heritability from twin studies include nonadditive as well as additive genetic variance (broad heritability), whereas adoption studies that involve first-degree relatives are largely limited to additive genetic variance (narrow heritability).^32^ If nonadditive genetic variance is the solution to the twin/adoption heritability difference for personality, it would imply that designs that do not involve MZ twins underestimate heritability caused by nonadditive genetic effects. In other words, twin studies do not overestimate heritability as compared to adoption designs; instead, adoption designs involving first-degree relatives estimate narrow heritability whereas twin studies estimate broad heritability. Although this means that nonadditive genetic variance again emerges as a good candidate for explaining the GCTA/twin heritability gap, this explanation goes against the pattern of MZ and DZ twin correlations for behavior problems in childhood in the present study, which shows no evidence for nonadditive genetic variance, as indicated earlier.

**Figure S1:**
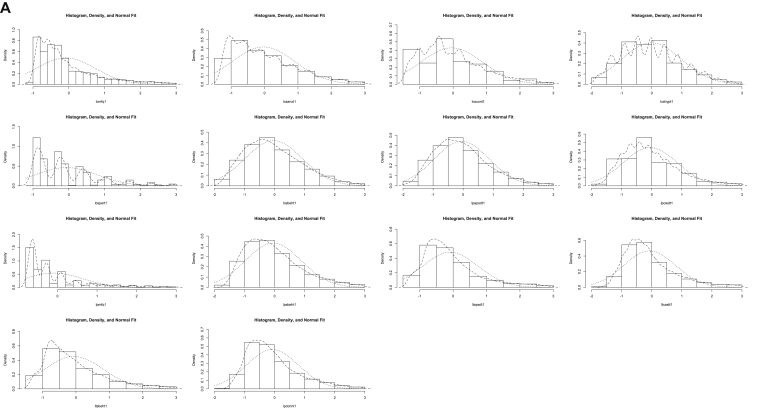

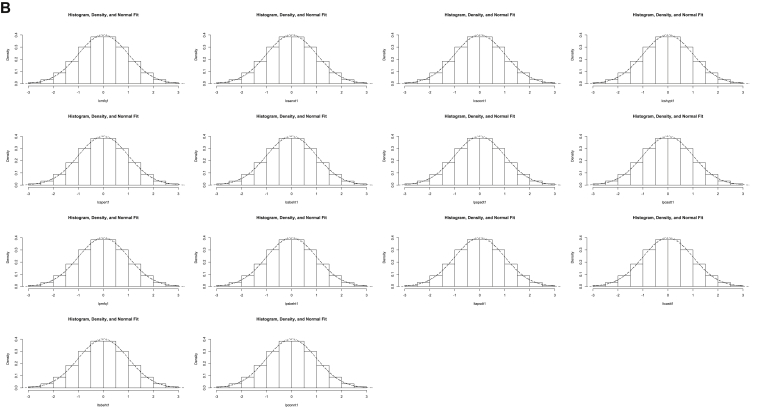
(A) Histograms of untransformed composite scales, (B) Histograms of composite quantile normalized scales. Note: As seen in Figure S1A, some of the composite scales are skewed, as is typical of behavior problem scales. However, Figure S1B shows that van der Wearden transformation (see Method) normalizes the scales. It is noteworthy that despite the considerable transformation of the distributions, the correlation between the untransformed and transformed scales are high (.0.90, 0.96, 0.96, 0.99, 0.92, 0.98, 0.98, 0.97, 0.86, 0.97, 0.94, 0.95, 0.94, 0.95, respectively), indicating that the transformation did not drastically disrupt the rank-order structure of the data. In the Results section of the text, we presented results for the transformed scales; however, as a further check on the effect of non-normality, we also compared genome-wide complex trait analysis (GCTA) and twin point estimates of heritability for the transformed and untransformed scales. We found that the largest GCTA heritability difference was 0.04 and the largest twin heritability difference was also 0.04. ‘lcmfq1’ = child self-rated Moods and Feelings Questionnaire (MFQ); ‘lcsanxt1’ = child self-rated Strengths and Difficulties Questionnaire (SDQ) Anxiety; ‘lcscont1’ = child self-rated SDQ conduct; ‘lcshypt1’ = child self-rated SDQ hyperactivity; ‘lcspert1’ = child self-rated SDQ peer problems; ‘lcsbeht1’ = child self-rated SDQ composite; ‘lpapsdt1’ = parent-rated APSD composite; ‘lpcstt1’ = parent-rated CAST composite; ‘lpmfq1’ = parent-rated MFQ composite; ‘lpsbeht1’ = parent-rated SDQ composite; ‘ltapsdt1’ = teacher-rated Antisocial Process Screening Device (APSD) composite; ‘ltcastt1’ = teacher-rated Childhood Asperger Syndrome Test (CAST) composite; ‘ltsbeht1’ = teacher-rated SDQ composite; ‘lpconnt1’ = parent-rated Conners attention-deficit/hyperactivity disorder (ADHD) composite.

Another methodological possible explanation of the low GCTA heritability estimates could be the skewed distributions, which are often found for measures of behavior problems. As shown in Figure S1, available online, some of the nontransformed distributions are skewed, but the transformed distributions, which were used in our analyses, are normal. To check on the possibility that the transformation could affect GCTA and twin estimates to different extents, we compared results for both methods using transformed and nontransformed scales and found little difference (see footnote in Figure S1, available online).

Overall, greater nonadditive genetic influence for behavior problems than for cognitive traits emerges as the leading candidate to explain the greater GCTA/twin heritability gap for behavior problems. The only problem with this explanation is that our twin results for behavior problems in children do not indicate nonadditive genetic effects, even though other twin studies of behavior problems in children and twin studies of adult personality point to some nonadditive genetic effects. In the absence of a more parsimonious explanation, we suggest that nonadditive genetic effects for behavior problems in childhood are masked by a general inflation of twin similarity for both MZ and DZ twins. One possibility is that this general twin inflation could be due to experiences that are shared by members of both MZ and DZ twin pairs. However, this possibility seems less implausible, because such shared experiences would seem likely to affect cognitive traits at least as much as behavior problems. A general inflation of twin similarity due to rating bias is another, more promising, possibility: a major difference between behavior problems and cognitive traits is that behavior problems are rated on questionnaires whereas cognitive traits are measured by tests, and ratings are inherently more prone to bias than tests. Such a general inflation of MZ and DZ twin correlations would mask nonadditive genetic variance because it would reduce the difference between MZ and DZ twin correlations. For example, let us suppose that the “true” MZ and DZ twin correlations were 0.3 and 0.1, respectively, suggesting some nonadditive genetic variance. Inflating both twin correlations by 0.1 would result in MZ and DZ correlations of 0.4 and 0.2, respectively, suggesting only additive genetic influence.

**Figure 3:**
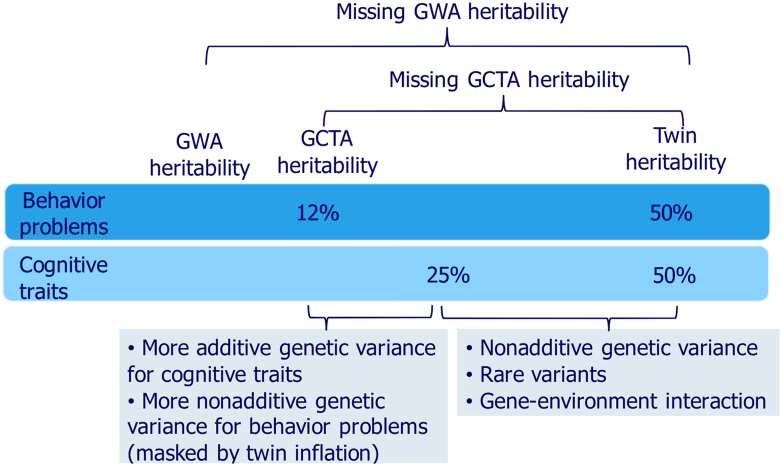
Missing genome-wide association (GWA) heritability and missing genome-wide complex trait analysis (GCTA) heritability for behavior problems and cognitive traits.

Figure 3 illustrates our hypothesis in the context of missing heritability. It introduces a second type of missing heritability. The familiar missing heritability is the extent to which the cumulative effect of all SNPs identified in GWA studies fall short of accounting for twin study heritability estimates. This could be called “missing GWA heritability” to distinguish it from “missing GCTA heritability,” which is the extent to which GCTA estimates fall short of accounting for twin study heritability estimates, and which sets the limit for GWA heritability because both GCTA and GWA are limited to detecting additive effects of common SNPs. We propose that twin studies do not overestimate heritability for behavior problems; rather, twin studies accurately detect nonadditive as well as additive genetic variance for behavior problems, but mostly additive genetic variance for cognitive traits. We do not suggest that all genetic variance for behavior problems is nonadditive, only that there is relatively more nonadditive genetic variance for behavior problems (masked by inflation of twin correlations for both MZ and DZ twins in the present study) and relatively more additive genetic variance for cognitive traits. If the heritability of behavior problems is about 50% and if about one half of the heritability is due to nonadditive genetic variance, whereas most of the genetic variance for cognitive traits is additive, this would explain the present results, as illustrated in Figure 3. If GCTA heritability for behavior problems were about one half of the GCTA heritability for cognitive traits (e.g., 12% vs. 25%, respectively, in Figure 3), the present study would have little power to detect it, as indicated by the standard errors in Figure 2.

In summary, we propose that a combination of 3 factors is responsible for the greater GCTA/twin heritability gap for behavior problems as compared to cognitive traits. First, nonadditive genetic variance is greater for behavior problems than for cognitive traits, reducing GCTA estimates for behavior problems relative to cognitive traits. Second, greater assortative mating for cognitive traits as compared to behavior problems produces more additive genetic variance for cognitive traits, which results in greater GCTA estimates and thus lowers the GCTA/twin heritability gap for cognitive traits but not for behavior problems. Third, the reason that our twin data do not indicate nonadditive genetic effects for behavior problems in childhood is that these nonadditive genetic effects are masked by inflated correlations for both MZ and DZ twins for ratings of behavior problems. Although this set of hypotheses is speculative, if true, it would suggest that GWA studies will find it more difficult to identify SNPs associated with behavior problems than with cognitive traits. It also suggests that nonadditive genetic variance might contribute importantly to the genetic architecture of behavior problems in childhood, and perhaps in adult personality and psychopathology as well.

## Figures and Tables

**Table S1:** Genome-Wide Complex Trait Analysis (GCTA) and Twin Genetic Estimates for Behavioral Problems Scales

Child self-reports	Twin Heritability							GCTA Heritability				
	A	SE	C	SE	E	SE	n/Pairs	V(G)	SE	V(e)	SE	n
MFQ (depressive symptoms)	0.38	0.06	0.09	0.05	0.53	0.02	2,683	0.00	0.12	1.00	0.12	2,698
SDQ Behavior Problems composite	0.44	0.06	0.09	0.05	0.47	0.02	2,668	0.00	0.12	1.00	0.13	2,690
Anxiety	0.41	0.03	0.00	0.01	0.59	0.02	2,668	0.02	0.12	0.99	0.13	2,687
Conduct	0.37	0.06	0.06	0.04	0.57	0.02	2,670	0.00	0.12	1.00	0.12	2,690
Hyperactivity	0.46	0.03	0.00	0.01	0.54	0.02	2,672	0.00	0.12	1.00	0.12	2,687
Peer problems	0.40	0.05	0.02	0.03	0.57	0.02	2,674	0.00	0.12	1.00	0.12	2,692

Parent ratings	Twin Heritability							GCTA Heritability				
	A	SE	C	SE	E	SE	n/Pairs	V(G)	SE	V(e)	SE	n
Conners ADHD composite	0.80	0.03	0.05	0.03	0.15	0.01	2,686	0.00	0.12	1.00	0.12	2,692
Hyperactivity-impulsivity	0.79	0.04	0.09	0.04	0.12	0.01	2,685	0.06	0.12	0.93	0.12	2,688
Inattention	0.79	0.01	0.00	0.01	0.21	0.01	2,687	0.00	0.12	1.00	0.12	2,693
APSD psychopathic symptoms composite	0.49	0.03	0.35	0.03	0.16	0.01	2,694	0.00	0.12	1.00	0.12	2,699
Callous-Unemotional	0.31	0.03	0.54	0.02	0.15	0.01	2,694	0.02	0.12	0.98	0.12	2,700
Impulsivity	0.66	0.04	0.16	0.04	0.19	0.01	2,687	0.00	0.12	0.99	0.12	2,697
Narcissism total	0.63	0.04	0.16	0.04	0.22	0.01	2,695	0.00	0.12	1.00	0.12	2,700
CAST autistic symptoms composite	0.73	0.04	0.06	0.03	0.22	0.01	2,688	0.09	0.12	0.91	0.12	2,694
Communication	0.76	0.03	0.01	0.02	0.23	0.01	2,689	0.00	0.12	1.00	0.12	2,692
Nonsocial	0.72	0.01	0.00	0.01	0.28	0.01	2,689	0.00	0.12	1.00	0.12	2,692
Social	0.71	0.02	0.00	0.01	0.29	0.01	2,687	0.06	0.12	0.94	0.12	2,693
MFQ (depressive symptoms)	0.71	0.04	0.06	0.03	0.24	0.01	2,680	0.00	0.12	1.00	0.12	2,688
SDQ Behavior Problems composite	0.60	0.04	0.21	0.03	0.19	0.01	2,687	0.00	0.12	1.00	0.12	2,692
Anxiety	0.61	0.04	0.02	0.03	0.38	0.02	2,683	0.00	0.12	1.00	0.12	2,687
Conduct total	0.55	0.04	0.22	0.04	0.23	0.01	2,685	0.00	0.12	1.00	0.12	2,691
Hyperactivity	0.78	0.01	0.00	0.01	0.22	0.01	2,687	0.00	0.12	1.00	0.12	2,691
Peer problems	0.78	0.01	0.00	0.01	0.22	0.01	2,685	0.16	0.12	0.84	0.12	2,690

Teacher ratings	Twin Heritability							GCTA Heritability				
	A	SE	C	SE	E	SE	n/Pairs	V(G)	SE	V(e)	SE	n
APSD psychopathic symptoms composite	0.61	0.05	0.02	0.03	0.37	0.02	1,901	0.15	0.16	0.85	0.16	2,129
Callous-unemotional	0.32	0.08	0.12	0.06	0.56	0.03	1,891	0.00	0.16	1.00	0.16	2,125
Impulsivity	0.63	0.02	0.00	0.01	0.37	0.02	1,898	0.24	0.16	0.76	0.16	2,120
Narcissism	0.65	0.02	0.00	0.01	0.35	0.02	1,904	0.50	0.16	0.50	0.15	2,128
CAST composite	0.48	0.04	0.00	0.02	0.52	0.02	1,896	0.00	0.16	1.00	0.16	2,120
Communication	0.50	0.03	0.00	0.01	0.50	0.02	1,899	0.00	0.15	1.00	0.16	2,121
Nonsocial	0.47	0.05	0.01	0.03	0.52	0.03	1,783	0.00	0.16	1.00	0.16	2,034
Social total	0.49	0.03	0.00	0.02	0.51	0.02	1,886	0.00	0.16	1.00	0.16	2,117
SDQ Behavior Problems composite	0.59	0.03	0.00	0.02	0.41	0.02	1,919	0.11	0.15	0.90	0.15	2,137
Anxiety	0.52	0.05	0.02	0.03	0.46	0.02	1,912	0.11	0.15	0.88	0.15	2,135
Conduct	0.54	0.02	0.00	0.01	0.46	0.02	1,921	0.26	0.15	0.73	0.15	2,137
Hyperactivity	0.57	0.03	0.00	0.01	0.43	0.02	1,925	0.05	0.15	0.95	0.15	2,138
Peer problems	0.54	0.04	0.00	0.02	0.46	0.02	1,918	0.00	0.15	1.00	0.16	2,139

Note: Composites are also presented in Figure 2. A = heritability estimate; ADHD = attention-deficit/hyperactivity disorder; APSD = Antisocial Process Screening Device; C = shared environment estimate; CAST = Childhood Asperger Syndrome Test; E = unique environment estimate; MFQ = Moods and Feelings Questionnaire; n = number of unrelated individuals with available data; n/Pairs = number of twin pairs with available data; SDQ = Strengths and Difficulties Questionnaire; SE = standard error; V(G) = variance explained by additive genetic factors.
